# 

**Published:** 1887-07

**Authors:** 


PAGE.

**ORIGINAL COMMUNICATIONS:**

- The Causes and Prevention of Infantile Diarrhœal Diseases (continued). By F. R. Campbell, A.M., M.D., . . . . . . . 529
- Abscess of Gall-Bladder. By Henry L. Elsner, M.D., Syracuse, N. Y., . . . . 538
- Pneumonia with Empyemia. By Henry L. Elsner, M.D., Syracuse, N. Y., . . . . 542

**CORRESPONDENCE:**

- The Metranoikter—Schatz's. Dr. M. Hartwig, . . . . . . . . . . . . . . 546

**SOCIETY REPORTS.**

- Erie County Medical Society, . . . . . 548

**MISCELLANY:**

- Clairvoyante Diagnosis, . . . . . . . 552
- The Causation of Malarial Fevers, . . . 553
- Latent Gonorrhœa, . . . . . . . . . 554
- Loss of Weight in Typhoid Fever, . . . 554

**SELECTIONS:**

- The Duration of Syphilogenic Capacity in Relation to Marriage, . . . . . . 555
- Another Remedy for Consumption, . . . 558
- On Horny Growth of the Penis, with Exhibition of a Remarkable Case, . . . . 559
- Stricture of the Female Urethra, . . . . 560
- Percutaneous Injection of Fluids into the Trachea, Extension of them into the Lung, and Effect of Them on the Lung and the Whole Organism, . . . . . . . . 560
- Chloride of Methyl: Its Uses in Neuralgias, 561
- Peptone Enemata, . . . . . . . . . 562
- Incomplete Abortion in Twin Pregnancy, . 562

**EDITORIAL:**

- The Board of Censors and Medical Education, . . . . . . . . . . . . . . 562
- Results of the Unilateral Removal of the Uterine Appendages, . . . . . . . 566

**REVIEWS:**

- Practical Lessons in Nursing. By Charles K. Mills, M.D., . . . . . . . . . . . 567
- Handbook of Materia Medica, Pharmacy and Therapeutics. By Samuel O. L. Potter, A.M., M.D., . . . . . . . . 567
- A Compend of Surgery for Students and Physicians. By Orville Horwitz, B.L., M.D., . . . . . . . . . . . . . 568
- A Compend of Electricity, and its Medical and Surgical Uses. By Charles F. Mason, M.D., and Charles H. May, M.D., . . . 568

### Index to Advertisements of Half a Page or Over.

PAGE.

